# Partial magnetic ordering in one-dimensional arrays of endofullerene single-molecule magnet peapods†

**DOI:** 10.1039/c8nr05386c

**Published:** 2018-10-04

**Authors:** Stanislav M. Avdoshenko, Fabian Fritz, Christin Schlesier, Aram Kostanyan, Jan Dreiser, Martina Luysberg, Alexey A. Popov, Carola Meyer, Rasmus Westerström

**Affiliations:** a Leibniz Institute for Solid State and Materials Research (IFW), 01069 Dresden, Germany; b Department of Physics, University Osnabrück, 49076 Osnabrück, Germany; c Peter Grünberg Institute (PGI-6), Forschungszentrum Jülich GmbH, 52425 Jülich, Germany; d Physik-Institut, Universität Zürich, Winterthurerstrasse 190, 8057 Zürich, Switzerland; e Swiss Light Source, Paul Scherrer Institute, 5232 Villigen PSI, Switzerland; f Ernst Ruska-Centre for Microscopy and Spectroscopy with Electrons, Forschungszentrum Jülich GmbH, 52425 Jülich, Germany; g Synchrotron Radiation Research, Lund University, 22100 Lund, Sweden

## Abstract

The magnetic ordering and bistability of one-dimensional chains of endofullerene Dy_2_ScN@C_80_ single-molecule magnets (SMMs) packed inside single-walled carbon nanotubes (SWCNTs) have been studied using high-resolution transmission electron microscopy (HRTEM), X-ray magnetic circular dichroism (XMCD), and *ab initio* calculations. X-ray absorption measurements reveal that the orientation of the encapsulated endofullerenes differs from the isotropic distribution in the bulk sample, indicating a partial ordering of the endofullerenes inside the SWCNTs. The effect of the one-dimensional packing was further investigated by *ab initio* calculations, demonstrating that for specific tube diameters, the encapsulation is leading to energetically preferential orientations of the endohedral clusters. Additionally, element-specific magnetization curves reveal a decreased magnetic bistability of the encapsulated Dy_2_ScN@C_80_ SMMs compared to the bulk analog.

The discovery of the giant magnetoresistance effect (GMR)^1,2^ in the late 1980s marks the start of the field of spintronics. A decade later came the first report on spin-dependent transport *via* a carbon nanotube (CNT)^3^ which opened the door for molecular spintronics. Studies of nanotube spintronics have demonstrated that spin valves with a large magnetoresistance ratio can be achieved by using magnetic La_(1–*x*)_Sr_*x*_MnO_3_ (LSMO) contacts.^4^ More recently, a study demonstrated that the need for magnetic contacts could be eliminated by magnetically coupling single-molecule magnets (SMMs) to the conducting channel of a carbon nanotube.^5^ In that study, a supramolecular spin valve was created with a spin-dependent transport solely determined by the magnetic properties of TbPc_2_ SMMs^6^ grafted to the outside wall of the single-walled CNT (SWCNT). The SMM-SWCNT device demonstrated the feasibility of an electronic readout of a single Tb^3+^ electronic and nuclear spin,^7^ a first step towards the application of SMMs in spintronic devices^8^ and quantum computing.^9^ An alternative route towards functionalizing CNTs is by filling the hollow interior with SMMs. The only published study of encapsulated SMMs has reported on the formation of amorphous structures of Mn_12_ clusters inside large diameter multi-walled CNTs.^10^ Endofullerenes encapsulating rare-earth based clusters constitute a new class of SMMs.^11–14^ Some SWCNTs have a diameter similar to that of the endofullerenes. The cylindrical interior of the SWCNT is thus ideally suited for encapsulating one-dimensional arrays of endofullerenes^15^ in so-called peapod structures.^16^

Here we report on the first study of one-dimensional arrays of SMMs packed into SWCNTs. The study was performed using high-resolution transmission electron microscopy (HRTEM), X-ray magnetic circular dichroism (XMCD), and *ab initio* calculations. The presented X-ray absorption spectroscopy (XAS) data demonstrates that the average orientation of the magnetic Dy_2_ScN-units of the encapsulated endofullerenes differs from the isotropic distribution in a bulk sample, indicating a partial ordering inside the nanotubes. A partial ordering is further supported by *ab initio* calculations which demonstrate that, depending on the specific tube diameter, the encapsulation results in energetically preferential orientations of the endohedral clusters. Nitride clusterfullerene SMMs have anisotropic magnetic clusters, and a preferred orientation inside the nanotubes implies a one-dimensional magnetic ordering with aligned molecular moments. The experimental and computational results thus demonstrate the feasibility of producing fullerene SMM-SWCNT hybrid devices consisting of well-protected and aligned molecular spin chains with possible applications in spintronics^8^ and quantum computation.^17,18^

Additionally, element-specific magnetization curves recorded using X-ray magnetic circular dichroism demonstrate a decreased average magnetic bistability of the encapsulated Dy_2_ScN@C_80_ endofullerenes.

## 1 Experimental and computational section

For the fabrication of the peapods, single-walled carbon-nanotubes (Carbon Solutions, Inc.) were chosen with a diameter distribution of 1.55 nm ± 0.1 nm to achieve one-dimensional filling. Raman spectra taken with a HeNe-Laser (*λ* = 632 nm), however, showed a radial-breathing mode (RBM) signal at 185 cm^−1^. This is a clear indication that metallic SWCNTs with diameters as small as 1.3 nm are present in the sample.^19^ The SWCNTs were oxidized in air for 20 min at 550 °C to create defects through which the fullerenes can enter the SWCNTs.^20^ Then the SWCNTs were sonicated in methanol for 10 min and deposited onto Cu TEM grids. These grids were used for high-resolution transmission electron microscopy as well as for the X-ray absorption measurements. For the filling, the grids were positioned in a glass container above the endofullerenes. Supercritical CO_2_ (50 °C, 150 bar) was used as a solvent, following the method of Khlobystov *et al.*^21^ To increase the filling yield the CO_2_ pressure was varied 40 times between 100 and 150 bar. After the process, the grids with the filled SWCNTs were washed with carbon disulfide to remove excess fullerenes. The HRTEM measurements were done using the spherical and chromatic aberration corrected FEI Titan 60–300 Ultimate (“PICO”)^22^ at an acceleration voltage of 80 kV. A slight underfocus was used to achieve high resolution. The Dy_2_ScN@C_80_ bulk sample was prepared by dissolving the endofullerenes in toluene and spray coated onto an aluminum sample holder. The X-ray absorption measurements were carried out at the X-Treme beamline^23^ of the Swiss Light Source. Absorption spectra were acquired by measuring the total electron yield (TEY) in the on-the-fly mode^24^ while applying a magnetic field parallel to the X-ray beam. The SQUID measurements were performed using a Quantum Design MPMS3 Vibrating Sample Magnetometer (VSM).

Static density functional theory (DFT) calculations were performed with the Vienna *ab initio* simulation package (VASP),^25,26^ which was used as optimization driver at the PBE level of theory.^27^ All optimizations were performed in Γ-point. During the optimizations, the energy-cut-off was set to 400 Ry and it correlates with pseudopotential choice,^26^ and all optimizations were run until residual forces became smaller than 0.005 Å eV^−1^. We used in-house Python scripts to analyze and visualize the results. Born–Oppenheimer molecular dynamics simulations were performed at the PBE-D/DZVP level using CP2K code.^28–30^ The velocity Verlet algorithm was employed with the time step of 0.5 fs and Nosé–Hoover thermostat (three chains, thermostat coupling constant 100 fs) set at 300 K. Overall integration time for each system was ~0.5 ns. Molecular structures, and BOMD trajectories were visualized using VMD package.^31^ Molecular theoretical XAS and XMCD spectra for the Dy *M*_4,5_-edge were simulated based on the point charge model implemented in MULTIX code.^32^ In these calculations, the Dy atoms assumed to be trivalent (Dy^3+^) and point charges of surrounding atoms are derived from molecular charge densities using Bader charge analysis for each molecule in the set of 144 isomers of Y_2_ScN@C_80_@CNT(12,8) (small).^33^ A set of semiempirical parameters was used to match experimental *M*_4_–*M*_5_ split (spin-orbital couplings of 0.95 for core, and of 0.85 for valence states) and energy spread of the *M*_5_ edge (on-site Coulomb of 0.75, see ref. 32 for details). To account for the experimental broadening, the theoretical spectra were convoluted with a Gaussian of *σ* = 0.5. The magnetic field strength and modeling temperature were set to the experimental values of 6.5 T and 2.0 K respectively. In all simulations, the X-ray beam is collinear to the magnetic field applied perpendicular to the tube-axis, see Fig. 5(a).

## 2 Results and discussion

**Fig. 1:**
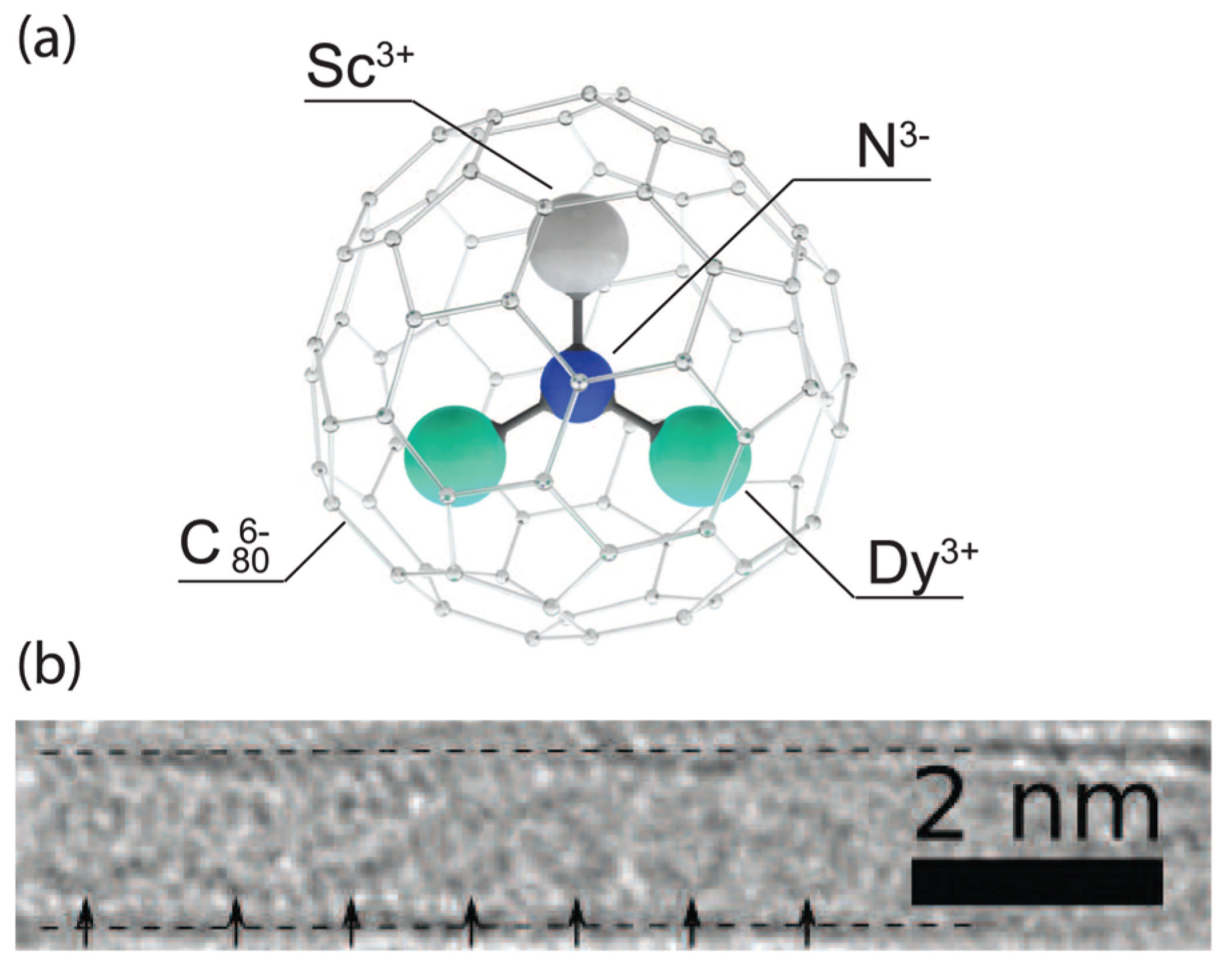
(a) Ball-and-stick-model of Dy_2_ScN@C_80_. (b) HRTEM image of a one-dimensional array of Dy_2_ScN@C_80_ endofullerenes inside a SWCNT. For clarification, the dashed lines give the position of the walls of the SWCNT, and the arrows point to the circular endofullerenes.

The HRTEM image presented in Fig. 1 shows the one-dimensional alignment of Dy_2_ScN@C_80_ endofullerenes inside a SWCNT of the sample recorded after the X-ray measurements. The C_80_ fullerene cages are visible as circles inside the SWCNT, demonstrating that there is no significant damage from the X-ray exposure. The image was taken using an exposure time of 0.5 s. Fullerenes and endohedral clusters appear a bit blurry due to their comparably fast movement (rotation) caused by the electron irradiation during the exposure.^34^

**Fig. 2:**
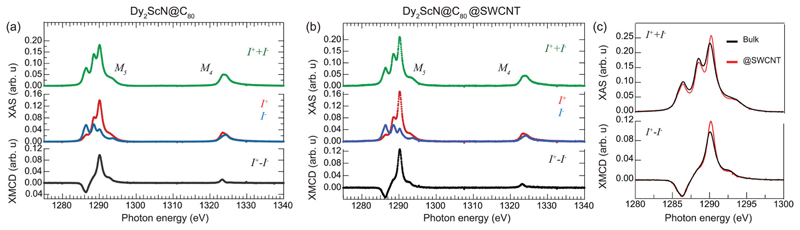
X-ray absorption spectra recorded using right (*I*^+^) and left (*I*^−^) circular polarized X-rays from samples consisting of (a) Dy_2_ScN@C_80_ endofullerenes in the bulk phase, and (b) encapsulated in SWCNTs. (c) A comparison of the normalized total absorption and XMCD spectra from the two systems. The temperature was set to 2 K, and an external magnetic field of 6.5 T was applied parallel to the X-ray beam and the surface normal of the samples. The data was normalized to the integrated total absorption (*I*^+^ + *I*^−^) over the displayed energy range.

Fig. 2 displays X-ray absorption spectra from a bulk and a peapod sample measured at a temperature of ~2 K and with an external magnetic field of 6.5 T applied along the X-ray beam. The spectra were recorded over the Dy *M*_4,5_-edge by exciting 3d core electrons to the 4f valence shell using right (*I*^+^) and left (*I*^−^) circular polarized photons. The difference in absorption between the two helicities and the resulting XMCD spectra (*I*^+^ – *I*^−^) contains information about the magnetic properties of the 4f valence shell of the absorbing Dy^3+^ ions. Applying a sum rule analysis^35,36^ to the displayed data provides absolute values for the spin 〈*S_z_*〉 and orbital 〈*L_z_*〉 moments and Table 1 shows the extracted values. The number of holes in the 4f shell was taken to be *n*_h_ = 5 and 〈*T_z_*〉 was calculated according to ref. 36. The ratio of the extracted orbital and spin angular moments is lower than, but close to the value of 5/2.5 = 2 expected from a ^6^H_15/2_ Hund’s ground state. The determined magnetic moments of $\mu =\left(\langle L_{z}\rangle +2\langle S_{z}\rangle \right)\frac{e}{2m}=4.7\mu_{\text{B}}$ for the bulk sample and *μ* = 5.2*μ*_B_ for the peapods, correspond roughly to half of the saturated magnetization of 10*μ*_B_ of an isotropic paramagnet with *J* = 15/2 and *g_J_* = 20/15. The reduced magnetic moments are a consequence of the anisotropic *m_J_* = 15/2 ground state with a magnetic easy-axis directed along the Dy–N bonds.^37^ Observing magnetic moments reduced by a factor of one-half is therefore expected for systems with an isotropic distribution of endohedral clusters.^11^ As was observed for Er_3_N@C_80_@SWCNTs,^15^ the comparable results from the bulk and peapod sample indicate that the anisotropic properties of the individual Dy^3+^ ions are preserved, further demonstrating that the endofullerenes remain intact after the encapsulation into SWCNTs and the subsequent X-ray exposure.

We return to the total absorption (*I*^+^ + *I*^−^) displayed in Fig. 2. The Dy *M*_5_ multiplet structure reflects the average orientation of the endohedral clusters with respect to the polarization vector of the X-rays.^38^ Comparing the total absorption at the Dy *M*_5_ edge from the bulk and the peapod sample in Fig. 2(c) reveals a difference in the multiplet structure. A deviation of ~9% is determined by integrating the absolute value of the difference between the two background subtracted and normalized spectra. The significant deviation from the isotropic bulk sample indicates that the endofullerenes are not completely randomly oriented inside the nanotubes. The magnitude of the XMCD signal is proportional to the projection of the magnetic moment of the absorbing Dy^3+^ ion onto the direction of the impinging X-rays. Because of the uniaxial anisotropy along the Dy–N bond, a deviation from the isotropic bulk sample should therefore also be observed in the XMCD signal. Comparing XMCD spectra in Fig. 2(c) and the extracted moments in Table 1 reveals a 22% larger maximum XMCD signal at the *M*_5_-edge and a 10% larger magnetic moment from the peapod sample, consistent with a partial ordering inside the nanotubes. It has been demonstrated that the torque exerted on the anisotropic Ho^3+^ moment in HoLu_2_N@C_80_ from an external magnetic field can lead to a thermally activated endohedral hopping motion that tends to align the cluster parallel to the field above a freezing temperature *T*_F_ ~ 55 K.^39^ In the present case, the system was cooled from room temperature to 2 K in zero field, and it is not expected that the applied field influences the partial ordering along the tubes. To understand the origin of the partial ordering we turn to *ab initio* calculations.

The fullerene cage C_80_-*I*_h_ is a quasi-spherical molecule, which gives rise to an almost isotropic distribution of cluster orientations inside the fullerene in the M_3_N@C_80_ molecule. As a result, at room temperature in solution, the M_3_N cluster is known to rotate quasi-freely inside the carbon cage. At the same time, chemical functionalization of the fullerene cage or supramolecular assembly (such as in co-crystals with Ni-octaethylporphyrin molecules^40^) will cause a partial freeze in the cluster rotation. That is, the cluster gains preferential orientation inside the cage governed by the external interactions. It is not known which effects are to be expected from a carbon nanotube on the M_3_N@C_80_ molecule inside. To address this problem, we performed extended computational studies of the Y_2_ScN@C_80_ molecule placed inside a carbon nanotube. It is well known that due to the “buried” nature of the f-shell, f-electrons rarely participate in chemical bonding. Therefore, the structural properties of lanthanide metallofullerenes can be well predicted by an analogous trivalent atom with close ionic radius. As ionic radii of Dy^3+^ and Y^3+^ are very close (0.91 Å and 0.90 Å, respectively^41^), the Y_2_ScN cluster closely matches the structural and dynamic properties of the Dy_2_ScN cluster inside the fullerene cage.

To understand the influence of the CNT encapsulation on the Dy_2_ScN cluster ordering and consequently the magnetic ordering, we have built the three model peapod systems Y_2_ScN@C_80_@CNT(14,7), Y_2_ScN@C_80_@CNT(12,8), and Y_2_ScN@C_80_@CNT(11,1). The two semiconducting chiral nanotubes were chosen to represent two different situations which the fullerene molecule may experience in the peapod. The inner diameter of the CNT(14,7) is 14.7 Å. This nanotube provides a tight fit of the Y_2_ScN@C_80_ molecule with comfortable π–π contact distance between fullerene and tube carbons of 3.2–3.4 Å. The system is therefore dubbed as **B** (“big”) in the following discussion. The inner diameter of CNT(12,8) is 13.6 Å, and the fullerene diameter has to be squeezed by 5% to fit the space inside the nanotube. The Y_2_ScN@C_80_@CNT(12,8) system is therefore dubbed as **S** (“small”). Importantly, the next smaller chiral CNT would be too small to fit the Y_2_ScN@C_80_ molecule in, and the next bigger one would be too roomy to have any effect on the Y_2_ScN cluster ordering. The only considered metallic tube **M** (“metal”) has a diameter of 14.9 Å which is similar to the CNT(14,7) and it provides a perfect fitting condition for the Y_2_ScN@C_80_ molecule inside. Having this nanotube included must help to understand the effect of near-zero-gap on the inner cluster ordering. Thus, this theoretical portfolio of CNTs covers experimentally expectable sizes (1.4–1.6 Å). In each system (**S**, **B**, and **M**), 144 conformational isomers were generated by 3D rotation of Y_2_ScN with respect to the cluster origin. The Euler’s angles increments were of δ*ϕ* = 60°, δ*θ* = 60°, and δ*ψ* = 40° which provides a representative structural cast, if not a sufficient sampling. All these systems were optimized at the PBE level of theory using the plane wave program VASP.

**Fig. 3:**
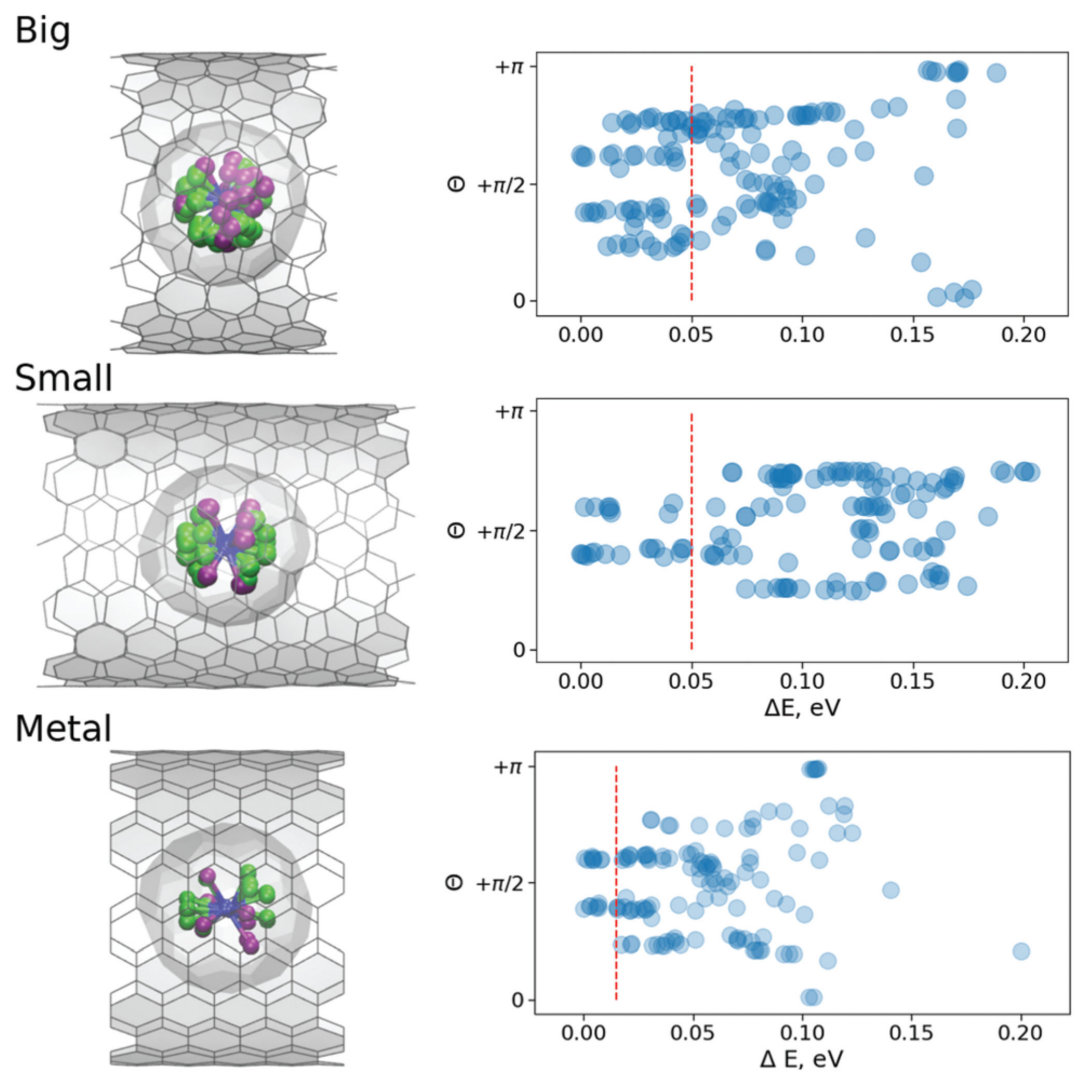
(Left) Superposed subset of optimized structures with the relative energy below the 0.05 eV thresholds (0.01 eV for the M-system), where Y-site is captured in light green and Sc-site in purple. (Right) Relative energy of different conformers of Y_2_ScNC_80_@CNT(14,7) (Big), Y_2_ScNC_80_@CNT(12,8) (Small) and Y_2_ScNC_80_@CNT(11,11) (Metal) systems as function of angle *θ* between the normal to the Y_2_ScN cluster plane and *z*-axis (aligned along the tube); the red dashed lines denote the energy threshold used to plot superposed structures in the left panel.

Fig. 3 shows the statistic of the optimized structures. Reportedly, in the free M_3_N@C_80_ molecule, there are three local minima for the cluster inside the fullerene cage (see ESI†). However, as one can see, the CNT confinement has produced multiple local minima. Moreover, the confinement has spread the energy spectra to higher energy end in case of **B** and **M** and even higher for the **S**-structures. Apparently, this is due to the fact that significant structural changes are prohibited by higher barriers in these structures and local nature of optimization procedure. Thus, only the closest minimum to the original structural guess has been located. The inner cluster/cage orientations in all these optimized structures are similar to those in the isolated molecule (see ESI†), but chemical compression by CNTs results in the spread of the relative energies (*i.e.* different orientations of the inner cluster with respect to the nanotube walls gain different energies). Thus, the encapsulation of the fullerene molecule inside the peapod results in the appearance of the energetically preferential orientations, and hence can lead to a partial ordering. Fig. 3 shows that the effect is stronger for the **S**-system than for the **M**-system or **B**-system. Moreover, as charge rearrangements (the charge transfer from CNT onto Y_2_ScNC_80_) in all systems are very similar we have concluded that the ordering is mostly driven by compression due to the confinement.

As Fig. 3 shows, the energy distributions in the peapods cover the range of 0.2 eV, which is twice as big as for the isolated fullerene molecule. The value corresponds to 8*k*_B_*T* for *T* = 300 K, the angle distribution at room temperature should be more or less uniform. However, as the sample in the XMCD experiment is cooled down to He temperature, a less uniform angular distribution can be expected. From Fig. 3 one can see that if the accessible energy window is limited to 0.05 eV, in the **S**-system the preferential orientation of the normal to the cluster plane is orthogonal to the CNT axis. Therefore, the **S**-system structures will likely produce XMCD response with the ordering effects. For **B**-system structures, the similar energy window covers a broader range of angles, and so, it is safe to assume that the XMCD response will be a powder-like. Very similar conclusions can be made based on the molecular dynamic (MD) studies discussed below.

**Fig. 4:**
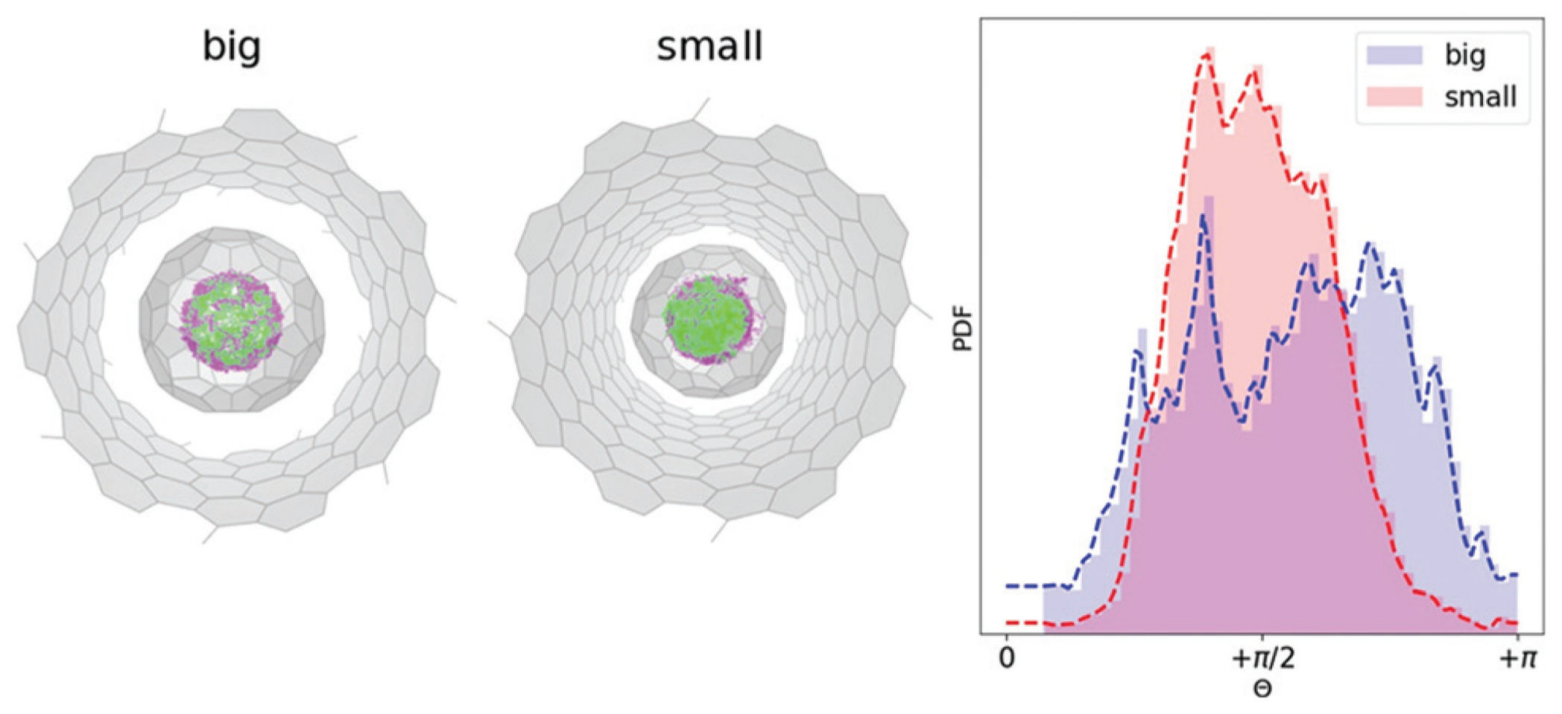
(Left) Superposed 144 Born–Oppenheimer MD trajectories (each propagated 5 ps) for Y_2_ScNC_80_@CNT(14,7) (big) and Y_2_ScNC_80_@CNT(12,8) (small). The Y and Sc atoms are captured in light green and purple respectively. (Right) Angle distribution *θ* (see Fig. 3) of the normal to the Y_2_ScN cluster-plane and *z*-axis (the CNT-axis) along joint 0.5 ns MD trajectory at *T* = 300 K.

Unfortunately, at experimentally relevant temperatures of XMCD measurements (~2 K) it would be hard to achieve any statistical significant integration of MD trajectories. At the same time, room temperature trajectories propagated for 5–10 ps would be sufficient to sample the local phase space. Moreover, if at room temperature the system would show any preferential geometric arrangements, it is reasonable to assume that after the cooling the preferential conformations would dominate the low-temperature phase. To study the dynamics of the endohedral cluster in the peapods we used the same 144 structures as initial guesses for 144 independent MD propagations with NVT ensemble at *T* = 300 K. Each geometry (in **S**- and **B**-system) was integrated for about 5–6 ps with 1 fs time step. This makes an overall integration time of 0.5 ns. Fig. 4 shows a superposition of all accessed geometries of the Y_2_ScN cluster in the **S**- and **B**-systems. Visually, the **B**-system has a more homogeneous spatial distribution of the positions of metal atoms (where Y-site is captured in light green and Sc-site in purple), and in the **S**-system the Y position is dominated in the front (which again points to a restricted rotation of the cluster in the **S**-systems). The histogram of the angle distribution between the normal to the cluster plane and the *z*-axis (*θ*) shows the same result in a numerical form (Fig. 4 (right)). According to Fig. 4, at room temperature, the **B**-system has a broader distribution of the *θ*-angles, while in the **S**-system the range of accessible angles is narrower and is biased towards *θ* = π/2.

**Fig. 5:**
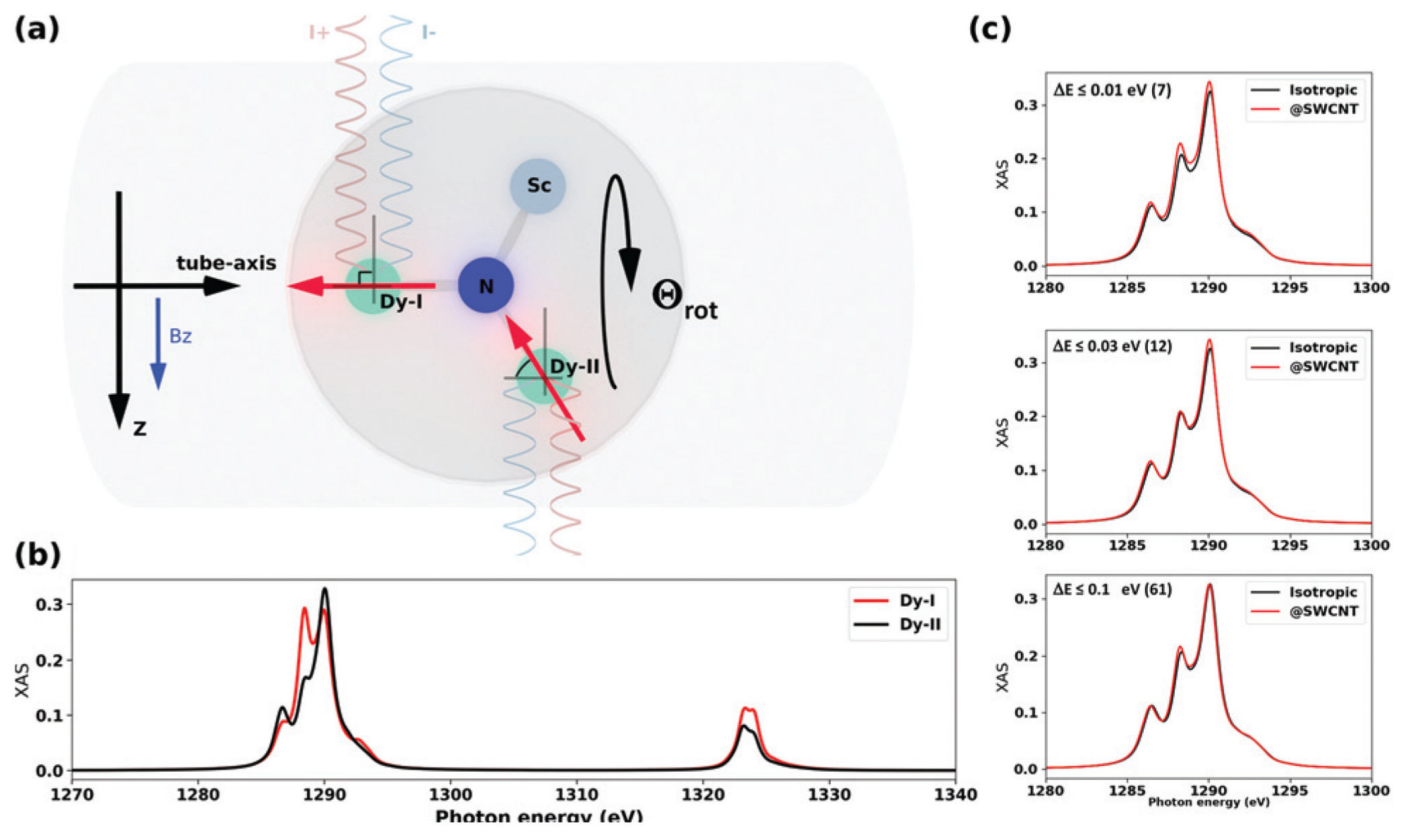
(a) Schematic representation of the peapods along the tube axis. The direction of magnetic field (*B_z_*) and direction of X-ray beams (*I*^+^ and *I*^−^) with respect to most probable positions of Dy-ions (Dy-I, Dy-II with the magnetic moments’ orientation in ground states *i.e. J_z_* = 15/2). (b) Theoretical XAS spectra for two Dy-sites as shown in (a), the model point charges *q*_N_ = −2 and *q*_Sc,Dy_ = +2 (these charges provide a correct structure of ^4^*I*_15/2_ multiplets). Note that rotation of the Dy_2_ScN cluster around (Dy-I)–N bond (*Θ*_rot_) would not change Dy-II spectrum. (c) Theoretical XAS for average overall 144 confirmation (isotropic) and well-optimized structures within relative energy window (≤0.01 eV (7), ≤0.03 eV (12), ≤0.1 eV (61)). The number in the round brackets indicates how many structures fall into the selected window (see Fig. 3).

At this point, one can justifiably conclude that the confinement of Dy_2_ScN@C_80_ inside a CNT is likely to orient the clusters so that one Dy-I within some accuracy is placed on the tube-axis while the second Dy-II is placed within the geometrical constraints (Dy–N–Dy angle 120°) with a freedom to rotate around the tube-axis (*Θ*_rot_ in Fig. 5(a) and 3). In our experimental setup, the incoming X-ray beam (as well as magnetic field *B_z_*) is always perpendicular to the tube-axis and due to cylindrical symmetry of this simplified problem, there are only two distinct XAS/XMCD spectra to be expected, with ground state magnetic moment perpendicular (90°) to the incoming X-ray beam and with 60° angle. These two spectra are shown in Fig. 5(b). Naturally, an average of these spectra would be different from an isotropic one due to a limited sampling. However, as our structural modelling indicates, positions of Dy-I and Dy-II would deviate from the proposed idealistic positions within a certain energy window. Fig. 5(c) shows a comparison between CNT-confined spectra and an isotropic spectrum (includes all 144 considered structures) for different sizes of the selected window. In fact, the average XAS spectrum obtained based on seven most stable structures (Fig. 5(c), energy Δ*E* ≤ 0.01 eV) reassembles closely experimental observations [Fig. 2(c)]. Having this window just 0.1 eV broad, theoretically, it would be impossible to tell the difference between @SWCNT and isotropic cases. This further suggests that the peapod system is likely to be under thermodynamic control and thus only most stable structures are allowed to form.

**Fig. 6:**
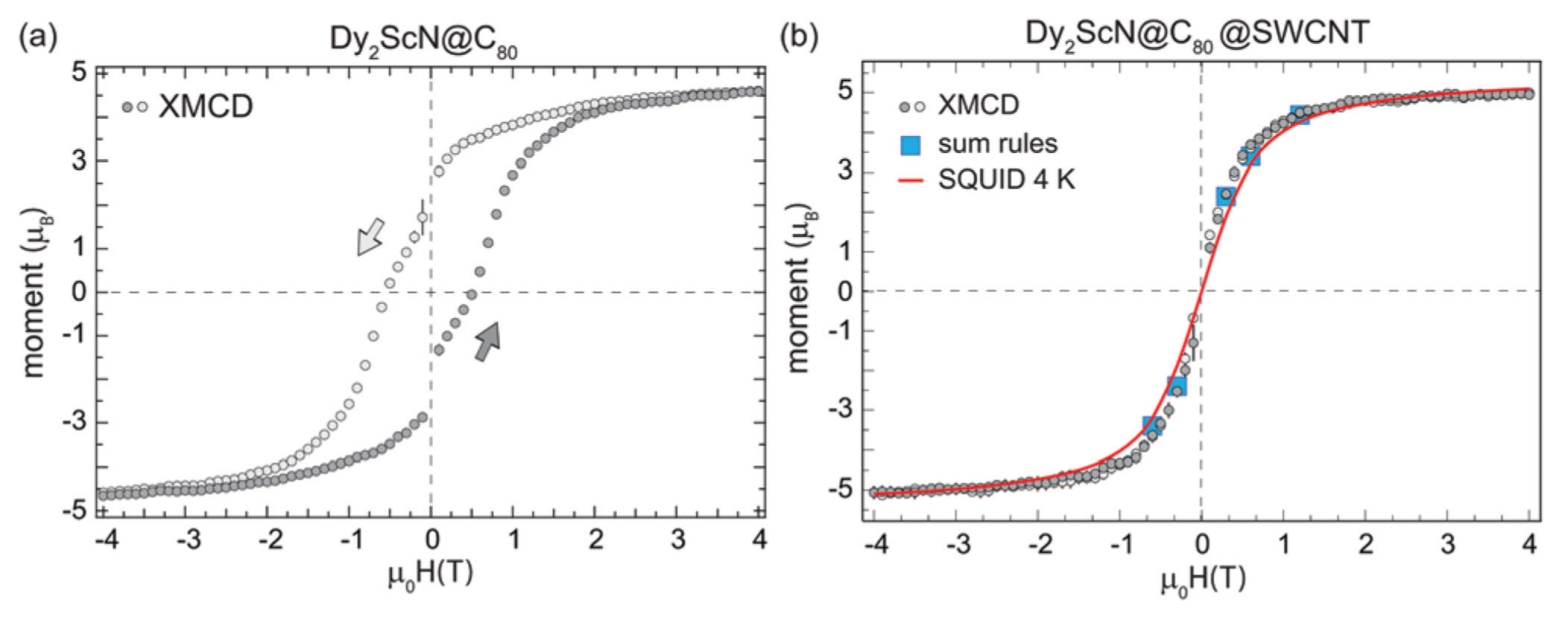
Element specific magnetization curves recorded at the Dy *M*_5_-edge with the temperature set to 2 K, a field sweep rate of 33 mT s^−1^, the X-ray beam parallel to the sample normal, and a photon flux of 5 × 10^10^ ph per s per mm^2^ from Dy_2_ScN@C_80_ endofullerens in the bulk phase (a) and encapsulated in SWCNTs (b). The magnetization curves correspond to the average of several independent measurements and the error bars shown as black bars are the standard deviation at each external magnetic field. The magnetization curves are scaled to the magnetic moments extracted by applying a sum-rule analysis to the XMCD data recorded at ±6.5 T (Table 1). The drop in magnetization at zero field in (a) is a consequence of the time of 30 s which it takes for the magnet to switch polarity. The 4 K magnetization curve in (b) was recorded using a SQUID with an effective field sweep rate of 0.4 mT s^−1^.

Recording the maximum XMCD signal at the Dy *M*_5_-edge while sweeping the magnetic field probes the response of the Dy^3+^ moments to external magnetic field changes. Fig. 6(a) displays an element-specific magnetization curve recorded from the bulk sample. The magnetization curve exhibits the characteristic low-temperature hysteresis of Dy_2_ScN@C_80_ with a significant remanent magnetization and coercive field.^37^ In strong contrast, the same measurement performed on the peapod sample yields a magnetization curve that, within the error bars, does not show a clear hysteresis. Hysteresis is observed in the system when the relaxation of the magnetization is slow compared to the measurement time. If instead, the system has time to relax to an equilibrium, hysteresis will not be observed and the magnetization curve will be reminiscent of a Brillouin function with a shape determined by the temperature.^37^ To verify that the absence of hysteresis in the peapod sample is not caused by a higher local temperature due to poor thermal conductance across the TEM grid, the data is compared to an equilibrium magnetization curve recorded with an average field sweep rate of 0.4 mT s^−1^ at 4 K from a bulk sample using a SQUID, see Fig. 6(b). This comparison is not taking into account the small anisotropy in the peapod sample which would translate into a slightly larger average moment and thereby modify the slope of the magnetization curve. However, a 10% increase in the effective magnetic moment would only correspond to a change in the temperature of a few percent. Therefore, the steeper slope observed in the magnetization curve recorded from the peapod sample in Fig. 6(b) demonstrates that there is no significant difference in the temperature between the two systems when performing the XMCD measurements, and there must be additional effects that are influencing the magnetic bistability of the encapsulated endofullerenes. Reduced magnetic bistability was observed in a sub-monolayer of Dy_2_ScN@C_80_ on a metal surface.^38^ Depending on the chirality, nanotubes can have zero bandgaps, and it is possible that encapsulating endofullerenes in metallic SWCNTs yield a similar effect. Substantial alteration of the magnetic properties has also been observed when depositing Fe_4_ SMMs on graphene.^42^ However, the nanotubes used in this study consist of a mixture of semiconducting and metallic tubes of different chiralities, and interaction with conducting electrons cannot be the only mechanism contributing to the observed decreased magnetic bistability. In the peapod sample, a fraction of the encapsulated endofullerenes could also be affected by dipole–dipole interaction with residue metallic catalyst nanoparticles. Though, such interactions should only have a minor influence on the total magnetic response of the system. The exposure to high photon flux can result in X-ray induced demagnetization.^43^ However, in the present case, the photon flux was the same for the two systems and this effect should not contribute to the observed differences between the two systems.

## 3 Conclusions

We have performed an HRTEM, XAS, and a computational study of one-dimensional arrays of Dy_2_ScN@C_80_ SMMs packed inside SWCNTs. X-ray absorption spectra recorded at the Dy *M*_5_-edge from the encapsulated Dy_2_ScN@C_80_ endofullerenes display deviation from that of an isotropic bulk sample which indicates a partial ordering of the endohedral clusters inside the nanotubes. Calculations performed on structurally analogous Y_2_ScN@C_80_ molecules packed into SWCNTs demonstrate that the encapsulation results in energetically preferential orientations of the endohedral clusters and that the ordering effect depends on the relative size of the fullerene cage and the diameter of the nanotubes. When compared to a bulk sample, element-specific magnetization curves recorded at ~2 K from SWCNTs filled with Dy_2_ScN@C_80_ SMMs reveal a decreased magnetic bistability and vanishing hysteresis for the encapsulated endofullerenes. In the case of endofullerene SMMs, a preferential orientation of the endohedral units implies alignment of the molecular anisotropy axis and magnetic ordering. Thus, the presented results demonstrate the feasibility of forming ordered and well protected one-dimensional spin chains that could possibly be addressed through the creation of SMM/SWCNT hybrid devices.

## Supplementary Material

†Electronic supplementary information (ESI) available. See DOI: 10.1039/C8NR05386C

SI

## Figures and Tables

**Table 1:** Expectation values of spin 〈*S_z_*〉 and orbital 〈*L_z_*〉 angular momentum operators extracted from a sum rule analysis of XMCD data measured at ±6.5 T and a temperature set to 2 K


Sample	〈*S_z_*〉 (ℏ)	〈*L_z_*〉 (ℏ)	〈*L_z_*〉/〈*S_z_*〉	〈*L_z_*〉 + 2〈*S_z_*〉 (ℏ)

Dy_2_ScN@C_80_-bulk	1.3 ± 0.1	2.1 ± 0.1	1.6 ± 0.1	4.7 ± 0.1
Dy_2_ScN@C_80_@SWCNT	1.4 ± 0.1	2.4 ± 0.2	1.7 ± 0.2	5.2 ± 0.2
