# Supplementary material for: Partial magnetic ordering in one-dimensional arrays of endofullerene single-molecule magnet peapods†
Source: Nanoscale. Author manuscript; Available in PMC 2019 Nov 8. (PMC6839967; doi:10.1039/c8nr05386c)
Supplement: SI [file EMS84815-supplement-SI.pdf]

Electronic Supplementary Material (ESI) for Nanoscale.
This journal is © The Royal Society of Chemistry 2018

ESI: Partial magnetic ordering in one-dimensional arrays of endofullerene single-molecule magnet peapods

-

July 3, 2018

Abstract

AThe magnetic ordering and bistability of one-dimensional chains of endofullerene $Dy_2ScN@C_{80}$ single-molecule magnets (SMMs) packed inside single-walled carbon nanotubes (SWCNTs) have been studied using high-resolution transmission electron microscopy (HRTEM), X-ray magnetic circular dichroism (XMCD), and *ab-initio* calculations. X-ray absorption measurements reveal that the orientation of the encapsulated endofullerenes differs from the isotropic distribution in the bulk sample, indicating a partial ordering of the endofullerenes inside the SWCNTs. The effect of the one-dimensional packing was further investigated by ab-initio calculations, demonstrating that for specific tube diameters, the encapsulation is leading to energetically preferential orientations of the endohedral clusters. Additionally, element-specific magnetization curves reveal a decreased magnetic bistability of the encapsulated $Dy_2ScN@C_{80}$ SMMs compared to the bulk analog

Keywords: SWCNT, peapods, single molecular magnets, XMCD, XAS, magnetodynamics, molecular dynamics, density functional theory. ■

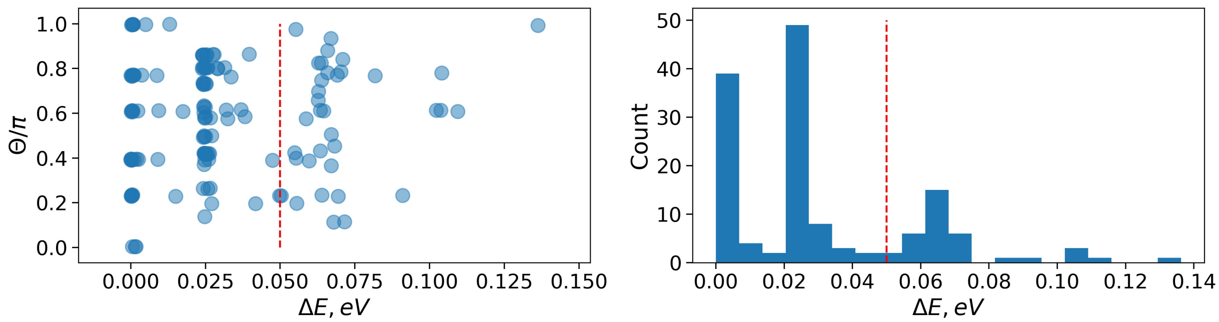

Figure S1: (left) Relative energy (VASP/PBE/PW) of different conformers of in the molecular Sc_3NC_{80}. There are only three main symmetry irreducible conformations, the fact is additionally illustrated by the energy histogram (right).

Geometries (geoms.tar.gz)

- The original structure $Dy_2ScN@C_{80}$ structre: **mol.xyz**
- 143 optimized structures and energies of the **B**-system : **b-opt.tar.gz**
- 143 optimized structures and energies of the **S**-system : **s-opt.tar.gz**

Scripts

Below we provide the examples of the computational scripts for VASP and CP2K codes (**Examples** 1, 2) and ASE python library based [ASE] generation scripts for the considered geometries (**Examples** 3, 4).

Example 1: Molecular dynamics CP2K settings:

```
    &XC
      &XC_FUNCTIONAL PBE
      &END XC_FUNCTIONAL
      &vdW_POTENTIAL
         DISPERSION_FUNCTIONAL PAIR_POTENTIAL
         &PAIR_POTENTIAL
            TYPE DFTD3
            PARAMETER_FILE_NAME dftd3.dat
            REFERENCE_FUNCTIONAL PBE
            CALCULATE_C9_TERM T
            R_CUTOFF 7.
         &END PAIR_POTENTIAL
      &END vdW_POTENTIAL
    &END XC
&MOTION
  &MD
    TEMPERATURE 300
    TIMESTEP 1.0
    STEPS 10000000
    ENSEMBLE NVT
    &THERMOSTAT
      &NOSE
        MTS 2
        LENGTH 3
        YOSHIDA 3
        TIMECON 100
      &END NOSE
    &END THERMOSTAT
  &END MD
&END
```

Example 2: VASP optimization settings:

```
PREC = Normal
ENMAX = 400
ISMEAR = 0 ; SIGMA = 0.1
IBRION = 1
NELM=200
LREAL=Auto
NSW = 400
EDIFFG = −0.005
ALGO = F
```

Example 3: Initial guess script part 1:

```
import ase.io as io
from ase.io import write, read
# Setup the system
fmol = read('./mol.xyz')
X=30.
Y=30.
Z=18.5689095
fmol. set_cell ([X,Y,Z])
fmol.pbc=[True,True,True]

fmol.center()

from ase.build import nanotube
tube = nanotube(12, 8)
X=30.
Y=30.
Z=18.5689095
tube. set_cell ([X,Y,Z])
tube.center()

comb=fmol+tube
write('tube_fmol.xyz', comb)
```

Example 4: Initial guess script part 2:

```
import ase.io as io
from ase.io import write, read
import os, sys
#system
mol = read('./tube_fmol.xyz')

cmol=mol.get_center_of_mass()
cage=mol[0:80]
cluster=mol[80:84]
rest=mol[84:len(mol)]
ll=0

for i in range(360/60):
  for j in range(360/60):
    for k in range(180/40):
      cluster . rotate_euler (phi=i*60., theta=j*60., psi=k*40., center=cmol)
      cnew=cage+cluster+rest
      pos=cnew.get_positions()
      ll=ll+1
      os.system("mkdir_%i" % ll)
      write('%i/mol.xyz' % ll, cnew)
```

[ASE] A. H. Larsen, J. J. Mortensen, J. Blomqvist, I. E. Castelli, R. Christensen, Marcin Duak, J. Friis, M. N. Groves, B. Hammer, C. Hargus, E. D. Hermes, P. C. Jennings, P. B. Jensen, J. Kermode, J. R. Kitchin, E. L. Kolsbjerg, J. Kubal, Kristen Kaasbjerg, S. Lysgaard, J. B. Maronsson, T. Maxson, T. Olsen, L. Pastewka, Andrew Peterson, C. Rostgaard, J. Schiotz, O. Schütt, M. Strange, K. S. Thygesen, Tejs Vegge, L. Vilhelmsen, M. Walter, Z. Zeng, K. W. Jacobsen, J. Phys.: *Condens. Matter*, 2017, DOI:10.1088/1361-648X/aa680e.
